# Impact of China’s National Centralized Drug Procurement Policy on pharmaceutical enterprises’ financial performance: a quasi-natural experimental study

**DOI:** 10.3389/fpubh.2023.1227102

**Published:** 2023-11-03

**Authors:** Zhixuan Sun, Xin Na, Shuzhen Chu

**Affiliations:** School of International Pharmaceutical Business, China Pharmaceutical University, Nanjing, China

**Keywords:** China’s National Centralized Drug Procurement, policy effect assessment, quasi-natural experiment, difference-in-difference, corporate financial performance

## Abstract

**Introduction:**

In China, the interest relationship between pharmaceutical enterprises and medical institutions has harmed the healthy development of pharmaceutical enterprises. In November 2018, the National Centralized Drug Procurement (NCDP) policy was published. The NCDP policy severs the interest relationship and significantly impacts on pharmaceutical enterprises’s financial performance.

**Methods:**

Using the implementation of China’s National Centralized Drug Procurement (NCDP) policy as a quasi-natural experiment, this study evaluated the impact of participation in the NCDP policy on pharmaceutical enterprises’ financial performance. We developed a difference-in-difference model to estimate the change in financial performance after NCDP implementation, based on financial data on Chinese listed pharmaceutical enterprises.

**Results:**

We found that the bid-winning enterprises’ financial performance significantly improved after participating in NCDP. This may be related to lower costs, market share expansion, and increased research and development investment by the bid-winning enterprises.

**Discussion:**

To further promote the high-quality development of pharmaceutical enterprises in China, the government should expand the variety of drugs on the NCDP list (NCDP drugs), while improving the drug patent protection system and the policies to support the bid-winning enterprises.

## 1. Introduction

The accessibility and affordability of medicines, a major challenge facing all countries worldwide, profoundly affects human health and development (1). Therefore, how to reduce drug prices and ease the burden on patients has become an issue for many countries, and China, which has long been plagued by high drug prices, is no exception (2, 3). Since the Chinese government decided to launch medical reforms in 2009, a series of policies such as the Centralized Drug Procurement System (implemented on a provincial basis) and the Zero Mark-up Drug Policy have been initiated, but drug prices have not been lowered to the expected degree because these policies have not delinked the medical institutions’ interests from drug prices (4, 5). Under the principle that medical institutions sell drugs for profit, pharmaceutical enterprises and medical institutions (e.g., hospitals) have formed an interest community, raising drug prices with the help of kickbacks and bribes. This is not only detrimental to patients’ interests, but it is also not conducive to the healthy development of pharmaceutical enterprises (6, 7). In these circumstances, Chinese pharmaceutical enterprises lack the motivation to innovate, invest most of their costs in marketing rather than in researching new drugs or optimizing production technologies, and produce drugs with excessive homogeneity (i.e., a large number of similar drugs can be substituted for each other, and there is a lack of quality drugs that stand out), which prevents efficient improvement of their financial performance. These issues have attracted the attention of researchers, and they have hypothesized that severing the interest relationship between pharmaceutical enterprises and medical institutions may efficiently increase corporate financial performance (8). The National Centralized Drug Procurement (NCDP) policy provides an opportunity to test this hypothesis.

To solve the problem of high drug prices and its consequences, in November 2018, the NCDP policy was published. This policy mandates that certain original drugs and generic drugs that have passed generic consistency evaluations (GCE) be procured in volume-based purchases in order to improve accessibility. In January 2019, the policy was first piloted in four municipalities and seven sub-provincial cities (thus it is called the “4 + 7” pilot). In May and July 2019, Fujian Province and Hebei Province joined the “4 + 7 pilot” respectively. In September 2019, the policy was extended to the remaining 25 provincial administrative regions in the Chinese mainland (thus it is called the “4 + 7” expansion). The “4 + 7” pilot and expansion were collectively referred to as the first round of NCDP. Since December 2019, another seven centralized drug procurement rounds, based on “volume-based procurement” and “volume–price linkage,” have occurred nationwide. Under the NCDP rules, the bid-winning enterprises are given 50–80% of the national market share for the next 1–3 years in exchange for lower prices (9). The NCDP policy involves drugs with high clinical usage and thus high procurement scope, which covers drugs for common outpatient diseases and major diseases basically (10). Thus, this is a major change in the pharmaceutical market. Under the NCDP policy, the National Healthcare Security Administration has gained strong regulatory power, thus severing the interest relationship between pharmaceutical enterprises and medical institutions. This inevitably has a significant impact on pharmaceutical enterprises (11).

Pharmaceutical enterprises, as drug producers, are the source of drugs and also important NCDP stakeholders (12). The change in the bid-winning enterprises’ financial performance before and after their participation in NCDP should inform the future direction of the NCDP policy and has important research value. After the NCDP implementation, the stock prices in the A-share market of the pharmaceutical industry experienced relatively large fluctuations (13). This phenomenon, indicating large variations in pharmaceutical enterprises’ financial performance, has attracted the interest of researchers, so the literature on the impact of the NCDP policy on pharmaceutical enterprises’ financial performance is growing (14).

Studies have assessed the impact of the NCDP policy on the pharmaceutical enterprises’ financial performance based on factors such as research and development (R&D), production and profitability. However, the studies differ in their conclusions. Some studies show that the NCDP policy had a positive impact on pharmaceutical enterprises’ financial performance due to increased R&D investment, innovation quality, and profit margins (15, 16). However, other research comes to opposite conclusions, finding that the NCDP policy decreased the enterprises’ profit margins (17). These differences indicate that this issue requires further research and discussion.

It is worth noting that most of the research samples in previous studies involve the “4 + 7” pilot centralized procurement period. However, at that time, the NCDP policy had just begun to be piloted, the procurement rules were imperfect, the institutional system was undeveloped, and data available for research were scarce (18, 19). In addition, some studies were limited to qualitative research, such as cases studies of certain enterprises. These factors may lead to biased results (13, 16). Therefore, this study was conducted based on the second round of NCDP (which represents a quasi-natural experiment) and aimed to explore the impact of the NCDP policy on financial performance using difference-in-difference (DID) models. Compared to previous research, the potential contributions and innovations of this study are as follows:

Based on input–output theory and dynamic capability theory, this study interpreted empirical results on the impact of the NCDP policy on the financial performance from two dimensions: procurement mechanism and policy orientation. This research perspective has rarely been seen in previous studies, so this study enriches the theoretical research and provides a comprehensive explanation of the impact of the NCDP policy on financial performance. Additionally, previous studies did not focus on the issue of the heterogeneous impacts of NCDP participation on bid-winning enterprises with different types of ownership. This study analyzed the changes in the financial performance of state-owned enterprises (SOEs) and non-SOEs after NCDP participation and provides potential explanations for the difference, which are of some practical significance.
The study was conducted based on the second round of NCDP, while most of the previous studies were based on the “4 + 7” pilot centralized procurement. After the “4 + 7” pilot and expansion, enterprises became more familiar with the NCDP rules and more rationally chose whether to participate in the second round of NCDP. In addition, the second round represents the first centralized procurement of drugs to occur simultaneously nationwide, and so the sample size has been significantly expanded (20). Therefore, this study has strong persuasive power.
Because the NCDP policy has not been in place for a long time, many of the previous studies are policy overviews and there are not many quantitative studies. This study systematically and comprehensively examined the impact of the NCDP policy on pharmaceutical enterprises’ financial performance using the DID model. To address the endogeneity issue and avoid the interference of unobservable factors, this study employed a variety of robustness tests, such as propensity score matching (PSM) and placebo test, to ensure that the results of the study are reliable.

The rest of this paper is organized as follows: the second section presents a literature review; the third section introduces the data collection process and the DID model; the fourth section presents the empirical test results and analysis of the results; and, finally, the discussion (including policy recommendations) and conclusion are provided in the fifth and sixth sections, respectively.

## 2. Literature review

### 2.1. Previous research on the NCDP policy

Unlike a single mandatory regulatory instrument or a market-based regulatory instrument, the NCDP policy, which has been thoroughly implemented in China, is a comprehensive regulatory instrument that contains both binding terms and various reward terms (11). Studies mainly cover (a) the evaluation of policy effectiveness and (b) its impact on various stakeholders. The former studies focus on the impact of the NCDP policy on drug prices and accessibility. For example, several studies conducted in different regions of China demonstrated the significant effects of the NCDP policy on lowering drug prices (especially regarding generics) and increasing the proportion of drugs that are affordable (especially regarding rural residents), and they analyzed the underlying reasons (21–25). The latter studies focus on the impact of the NCDP policy on patients, medical institutions, and pharmaceutical enterprises. Several researchers have explored patients’ health care expenditures and attitudes toward the NCDP policy after its implementation, as well as the use of NCDP drugs in public medical institutions (26–34). However, few researchers have focused on the impact of the NCDP policy on pharmaceutical enterprises, particularly on their financial performance, which is the focus of this study.

### 2.2. Previous research on enterprises’ financial performance

Financial performance is a widely used indicator in corporate performance research. In addition to maximizing profits, enterprises should also consider their responsibilities to society, the environment and the government (35). In turn, an enterprise’s financial performance is affected not only by the enterprise itself and the industry, but also by various factors related to society, the environment and policies (36). Many researchers have assessed the impact of different policies on enterprises’ financial performance, but the impact is not always positive due to differences in policy objectives and policy specific practices (37).

### 2.3. NCDP policy and pharmaceutical enterprises’ financial performance

According to the resource dependence theory and input–output theory, differences in the resources invested in the operation of an enterprise lead to different outputs, which creates differences in financial performance and profitability (38). A good input–output relationship is closely related to a high level of financial performance. However, as the economic environment is in a state of dynamic change (e.g., due to enactment of new policies or the occurrence of major public health events), enterprises’ input–output relationships are not static. In dynamic capability theory, dynamic capability refers to the adaptive ability of an enterprise to reintegrate existing resources and form new input–output relationships in a dynamically changing environment (39, 40). This study focuses on whether, under the exogenous impact of the NCDP policy, pharmaceutical enterprises have utilized their dynamic capabilities and how their financial performance has been affected.

Although previous research has provided some insight, studies on the impact of the NCDP policy on pharmaceutical enterprises’ financial performance are still quite scarce, and most of them focus on theoretical analysis and lacking empirical studies (17, 41, 41). The implementation of the NCDP policy constitutes a quasi-natural experiment that can help to identify the impact of the policy on pharmaceutical enterprises’ financial performance (31). Using the data on listed companies in the China Stock Market & Accounting Research Database (CSMAR), we systematically and comprehensively examined the impact of the NCDP policy on pharmaceutical enterprises’ financial performance using the DID method, the PSM method, and other methods.

## 3. Materials and methods

### 3.1. Methodology

The DID model has been widely used to evaluate the effect of policy implementation. In recent years, several researchers have used the DID model to assess the impact of different policies on enterprises’ financial performance (37, 42, 43).

The second round of NCDP involved 78 bid-winning pharmaceutical enterprises and 32 drugs (according to the procurement document and the list of NCDP drugs published by China’s Joint Procurement Office), which provided a good quasi-natural experiment for use in this study (44). The study sample consisted of 174 A-share listed pharmaceutical enterprises on the Shanghai and Shenzhen stock exchanges. Of these enterprises, 20 bid-winning enterprises that participated in the second round of NCDP constituted the experimental group and the other 154 enterprises constituted the control group. By comparing the changes in the financial performance of the experimental and control groups after the second round of NCDP, the DID model assessed the impact of the NCDP policy on the pharmaceutical enterprises’ financial performance.

The DID model was constructed as follows:


$$\mathrm{performanc}e_{\mathrm{it}}=\alpha_{0}+\alpha_{1}DID_{\mathrm{it}}+\alpha_{2}X_{\mathrm{it}}+\mu_{i}+\lambda_{t}+\varepsilon_{\mathrm{it}}$$

The dependent variable, $\mathrm{performanc}e_{\mathrm{it}}$, indicates the financial performance of enterprise i in quarter t. The core explanatory variable, $DID_{\mathrm{it}}$, is an NCDP policy dummy variable, which was assigned according to the NCDP document. It was equal to one if the enterprise belonged to the experimental group and the time point was after the second round of NCDP, and zero otherwise. $\alpha_{1}$ is the core estimation parameter that indicates the effect of the NCDP policy on pharmaceutical enterprises’ financial performance. If $\alpha_{1}$ is positive, it indicates that the implementation of the NCDP policy improved pharmaceutical enterprises’ financial performance, whereas a negative value indicates an inhibitory effect. $X_{\mathrm{it}}$ is a set of control variables that may affect the pharmaceutical enterprises’ financial performance. $\mu_{i}$ and $\lambda_{t}$ denote enterprise fixed effects and quarter fixed effects, respectively. $\varepsilon_{\mathrm{it}}$ is the random error term.

### 3.2. Data

A-share listed pharmaceutical enterprises on the Shanghai and Shenzhen stock exchanges operating during the second quarter of 2018 to the fourth quarter of 2021 were selected as the study sample. To ensure the reliability and stability of the sample data, the following enterprises were excluded: (1) enterprises with consecutive losses (ST and ST* companies); (2) enterprises with considerable missing data on the research variables; (3) enterprises that were first listed after the second round of NCDP (fourth quarter of 2019); and (4) enterprises that produce medical devices, medical consumables, veterinary drugs, or pharmaceutical excipients. As a result, 174 enterprises (20 in the experimental group [bid-winning enterprises that participated in the second round of NCDP] and 154 in the control group) were finally retained. Details of the 174 enterprises are given in the Supplementary Material.

Data on corporate characteristics (such as corporate financial indicators and equity data) were obtained from CSMAR. For the small number of missing values, linear interpolation was used to fill in the gaps.

The stock overall growth score (OGS), provided by CSMAR, was used as the dependent variable $\left(\mathrm{performanc}e_{\mathrm{it}}\right)$ to assess financial performance. OGS incorporates the growth rate of earnings, net assets, main business income, and operating cash flow, which can reflect financial performance in a more reasonable and comprehensive way compared to the return on common stockholders’ equity (ROE) or TobinQ, which were the main indicators used in previous studies.

Based on previous studies on financial performance (45, 46), the following eight enterprise characteristics were selected as control variables: (1) transaction cost (TC), which is equal to the ratio of selling expenses to revenue from the primary business; (2) volatility, which is measured by taking the natural logarithm of the stock’s return over the last 250 trading days; (3) financial leverage (Lev), which is the ratio of total liabilities to total assets; (4) return on assets (ROA), which is the net income divided by the total assets; (5) enterprise size (Size), which is measured by taking the natural logarithm of the book value of total assets; (6) book-to-market ratio (BM); (7) ownership concentration (Top10), which can reflect the enterprise’s shareholding structure and can be measured by the shareholding percentage of the top ten shareholders; and (8) earnings per share (EPS) of the company’s stock. These variables and the data were obtained by manually screening the CSMAR quarterly reports. A summary of the abbreviations and definitions of all variables are shown in Table 1. Descriptive statistics for each variable are presented in Table 2.

**Table 1:** Summary of abbreviations and definitions of variables.

	Variable	Abbreviation	Definition
Dependent variable	Overall growth score	OGS	Sum of growth rate of earnings, net assets, main business income, and operating cash flow/4
Explanatory variables	DID	DID	NCDP policy dummy variable
Control variables	Transaction cost	TC	Selling expenses/revenue from primary business
	Volatility	Volatility	Log (rate of return on the stock over previous 250 trading days)
	Financial leverage	Lev	Total liabilities/total assets
	Return on assets	ROA	Net income/total assets
	Enterprise size	Size	Log (book value of total assets)
	Book-to-market ratio	BM	Book value/market value
	Ownership concentration	Top10	Number of shares held by top 10 largest shareholders/total shares
	Earnings per share	EPS	Earnings per share of the company’s stock

**Table 2:** Descriptive statistics for variables.

Variable	Mean	Min	Max	Standard deviation
OGS	0.267	−10.153	17.884	1.478
TC	0.297	0.006	1.175	0.181
Volatility	0.420	0.151	0.951	0.105
Lev	0.311	0.011	0.940	0.161
ROA	0.040	−0.847	0.451	0.058
Size	22.160	19.518	25.260	1.019
BM	0.545	0.040	1.275	0.228
Top10	56.986	19.830	91.410	13.958
EPS	0.580	−3.581	11.609	1.034

## 4. Results

### 4.1. Results of main DID regression

The regression results regarding the effect of the NCDP policy on pharmaceutical enterprises’ financial performance are shown in Table 3. Model (1) controlled for enterprise fixed effects and quarter fixed effects, and the regression coefficient of DID was 0.519, which was significant at the 1% level. This indicates that the financial performance of the enterprises participating in NCDP was significantly higher than that of the non-participating enterprises. Model (2) was based on model (1) with added control variables to improve the goodness-of-fit of the model. The coefficient of DID in model (2) was 0.507, which was significant at the 1% level.

**Table 3:** Impact of NCDP participation on financial performance: main regression results.

Variable	Dependent variable: OGS	
	Model (1)	Model (2)
DID	0.519*** (0.112)	0.507*** (0.107)
TC		−0.939*** (0.295)
Volatility		−0.195 (0.246)
Lev		−2.013*** (0.326)
ROA		−1.142*** (0.418)
Size		1.212*** (0.114)
BM		−0.076 (0.213)
Top10		0.027*** (0.005)
EPS		0.124*** (0.037)
Constant	0.235*** (0.019)	−27.125*** (2.441)
Enterprise fixed effects	Yes	Yes
Quarter fixed effects	Yes	Yes
adj. R^2^	0.618	0.657

Robustness standard errors clustered at the enterprise level are in parentheses.*, **, and *** indicate significance at the 10, 5, and 1% levels, respectively.

These empirical results indicate that NCDP participation has a significant positive impact on corporate financial performance. In theory, NCDP participation may cause enterprises to experience difficulties in drug production capacity and supply security. However, NCDP policy provisions such as 50–80% market share guarantee and upfront reimbursement can offset the costs required for active pharmaceutical ingredients (API) procurement and capacity expansion. In addition, NCDP participation encourages R&D innovation, which can be reflected in stock market fluctuations and improved financial performance.

### 4.2. Robustness tests

#### 4.2.1. Parallel trend test

**Figure 1:**
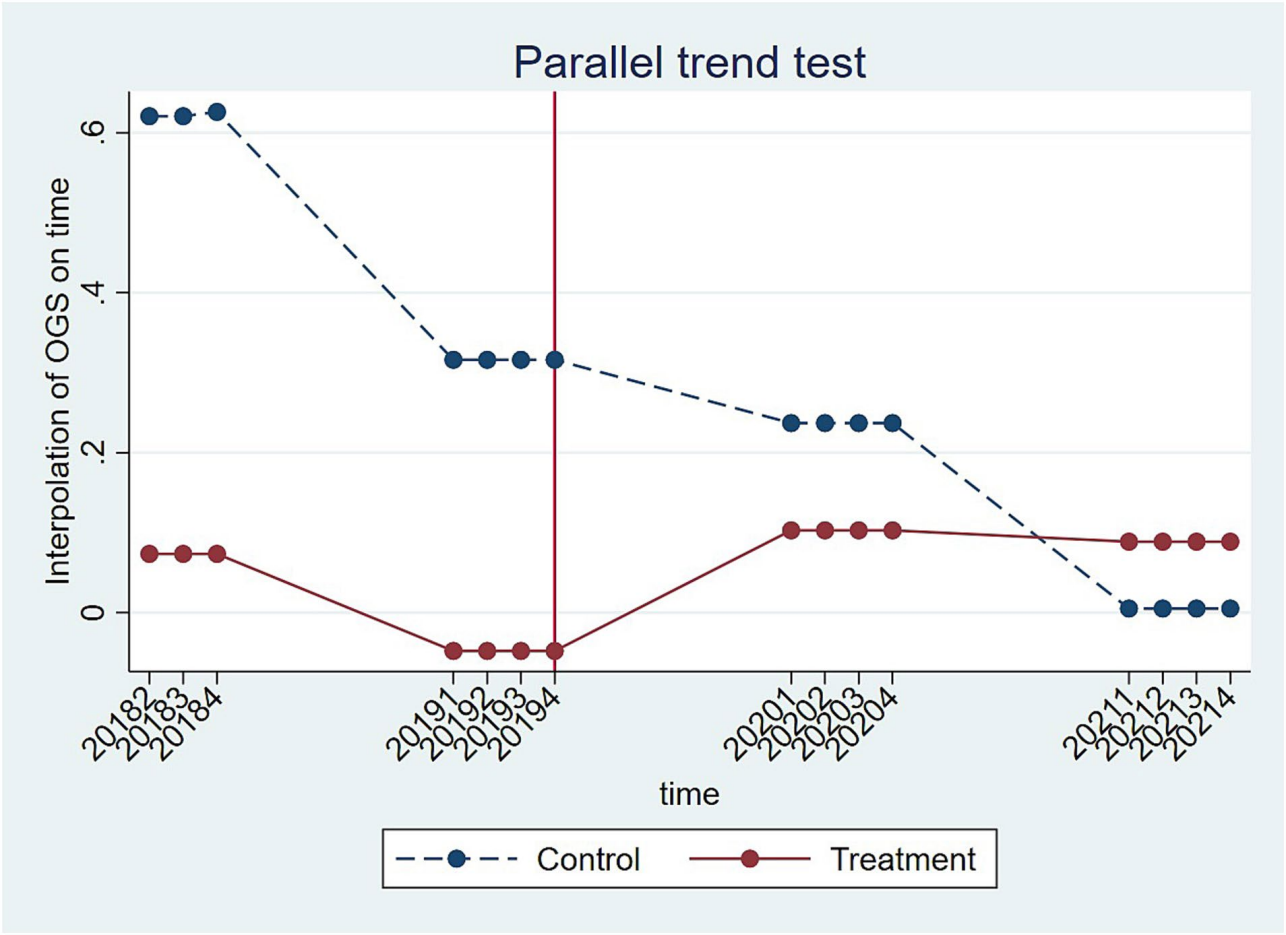
Trends in financial performance of enterprises in the experimental and control groups from the second quarter of 2018 to the fourth quarter of 2021.

To use the DID model, the assumption that there is a common trend between the experimental and control groups must be valid (47). In other words, without the NCDP policy, the trends in overall financial performance of the enterprises in the experimental and control groups must not differ systematically over time. Therefore, a parallel trend test was used to examine the time trends in the financial performance of the enterprises in the experimental and control groups in the first six and last eight quarters of the study period (i.e., second quarter of 2018 to fourth quarter of 2021), with the seventh quarter (i.e., fourth quarter of 2019, December 2019) being when the NCDP policy was extended across the whole country. The result of the test is shown in Figure 1. The curves of the experimental and control groups are nearly parallel until the fourth quarter of 2019. However, after NCDP implementation, the curve of the experimental group shows a clear upward trend, while the curve of the control group shows the exact opposite downward trend. This result demonstrates that the NCDP implementation can be considered to represent a quasi-natural experiment and preliminarily validates the robustness of the DID model regression results.

#### 4.2.2. PSM-DID

Although the DID model used in the main regression dealt with the endogeneity problem, it is difficult to solve the sample bias problem (48). As different enterprises often have different characteristics, the results of the parallel trend test were not fully convincing. Thus, the PSM approach was used and a PSM-DID model was constructed to assess the net effect of the NCDP policy by addressing the sample bias problem and controlling for unobservable and non-time-varying differences among enterprises (49, 50).

**Figure 2:**
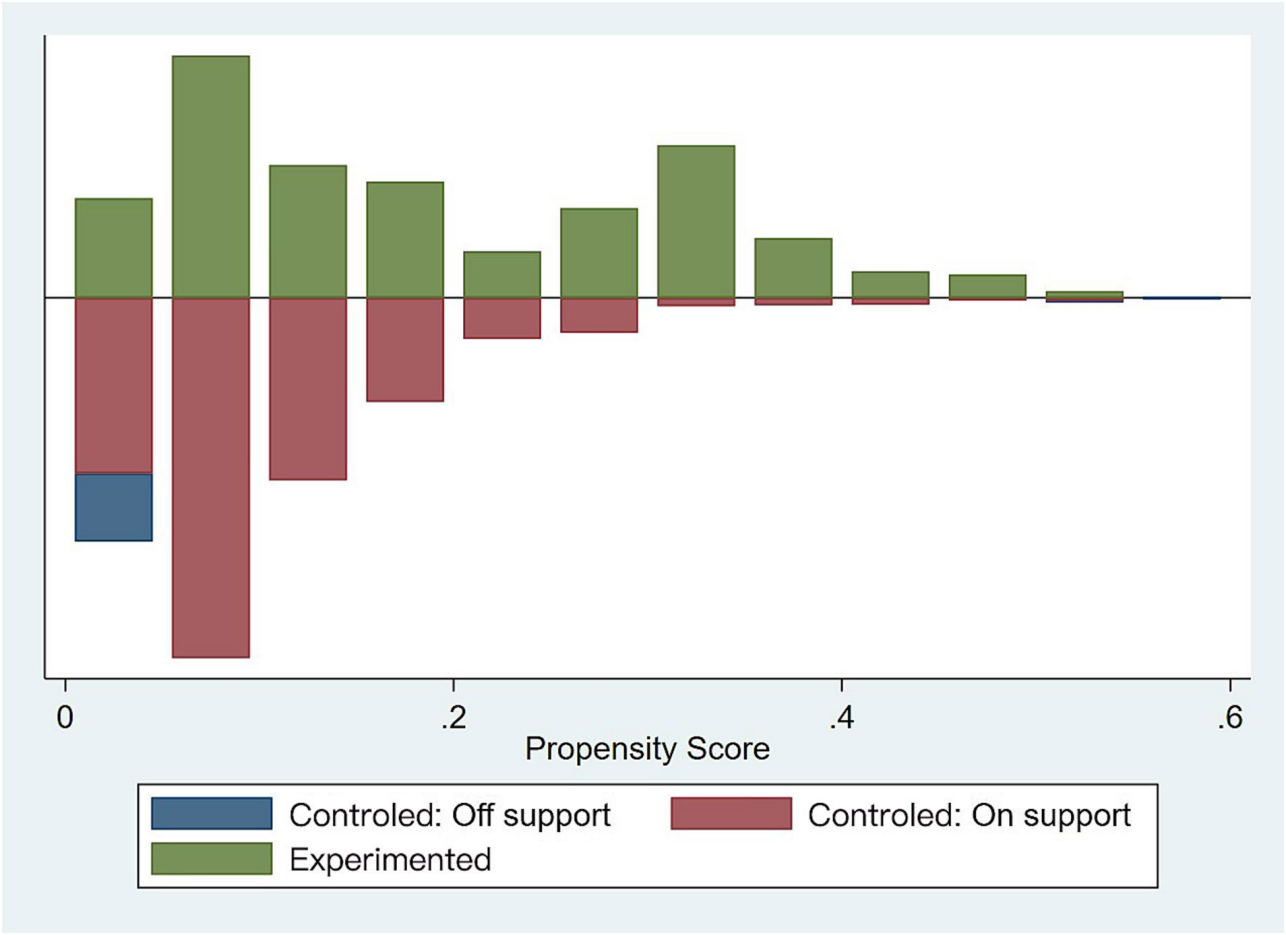
Distribution of propensity scores in the experimental and control groups.

First, nearest neighbor matching was used to estimate propensity scores by combining enterprise characteristic variables (i.e., eight control variables) and logit regressions to select control group enterprises for the experimental group enterprises, thus eliminating the differences between the two groups. The standard deviations of most of the variables were much lower after matching, with the values for all variables except Top10 being <10% (Table 4). This indicates that the sample bias was reduced and there were no significant differences between the experimental and control groups. Figure 2 shows the distribution of propensity scores in the experimental and control groups, indicating high inclusion regarding the matching.

**Table 4:** Suitability test (common supporting hypothesis).

Variable	Unmatched/Matched	Mean treated	Control	% bias	% reduct|bias|
TC	U	0.298	0.297	1.0	−204.7
	M	0.298	0.293	3.0	
Volatility	U	0.424	0.419	4.6	95.7
	M	0.424	0.424	−0.2	
Lev	U	0.395	0.300	57.1	84.8
	M	0.395	0.409	−8.7	
ROA	U	0.035	0.041	−10.7	81.4
	M	0.035	0.033	2.0	
Size	U	22.677	22.090	53.8	98.8
	M	22.677	22.670	0.6	
BM	U	0.548	0.545	1.3	−462.8
	M	0.548	0.564	−7.1	
Top10	U	57.115	56.969	1.0	−1009.1
	M	57.115	58.725	−11.5	
EPS	U	0.433	0.599	−19.0	99.0
	M	0.433	0.435	−0.2	

Subsequently, the matched samples were subjected to DID estimation, with the estimates of the PSM-DID model being presented in column (1) of Table 5. The regression coefficient of DID was 0.449, which was similar to the main DID regression results in Table 3 (model (1): 0.519; model (2): 0.507) and which remained significant at the 1% level.

**Table 5:** Impact of NCDP participation on financial performance: PSM-DID regression results.

Variable	PSM-DID model (1)	PSM-DID model (2)
DID	0.449*** (0.139)	0.802*** (0.109)
Control variables	Yes	Yes
Enterprise fixed effects	Yes	Yes
Quarter fixed effects	Yes	Yes
adj. R^2^	0.654	0.030

Finally, to further verify the robustness of the main DID regression results, the nearest neighbor matching was replaced by kernel matching to estimate the propensity scores and the DID regression was performed again. The estimated results are shown in column (2) of Table 5. The regression coefficient of DID was 0.802, which was significant at the 1% level. The results of the PSM-DID models support the results of the main DID models and validate the reliability of the finding that the NCDP policy improved pharmaceutical enterprises’ financial performance.

#### 4.2.3. Placebo test

**Figure 3:**
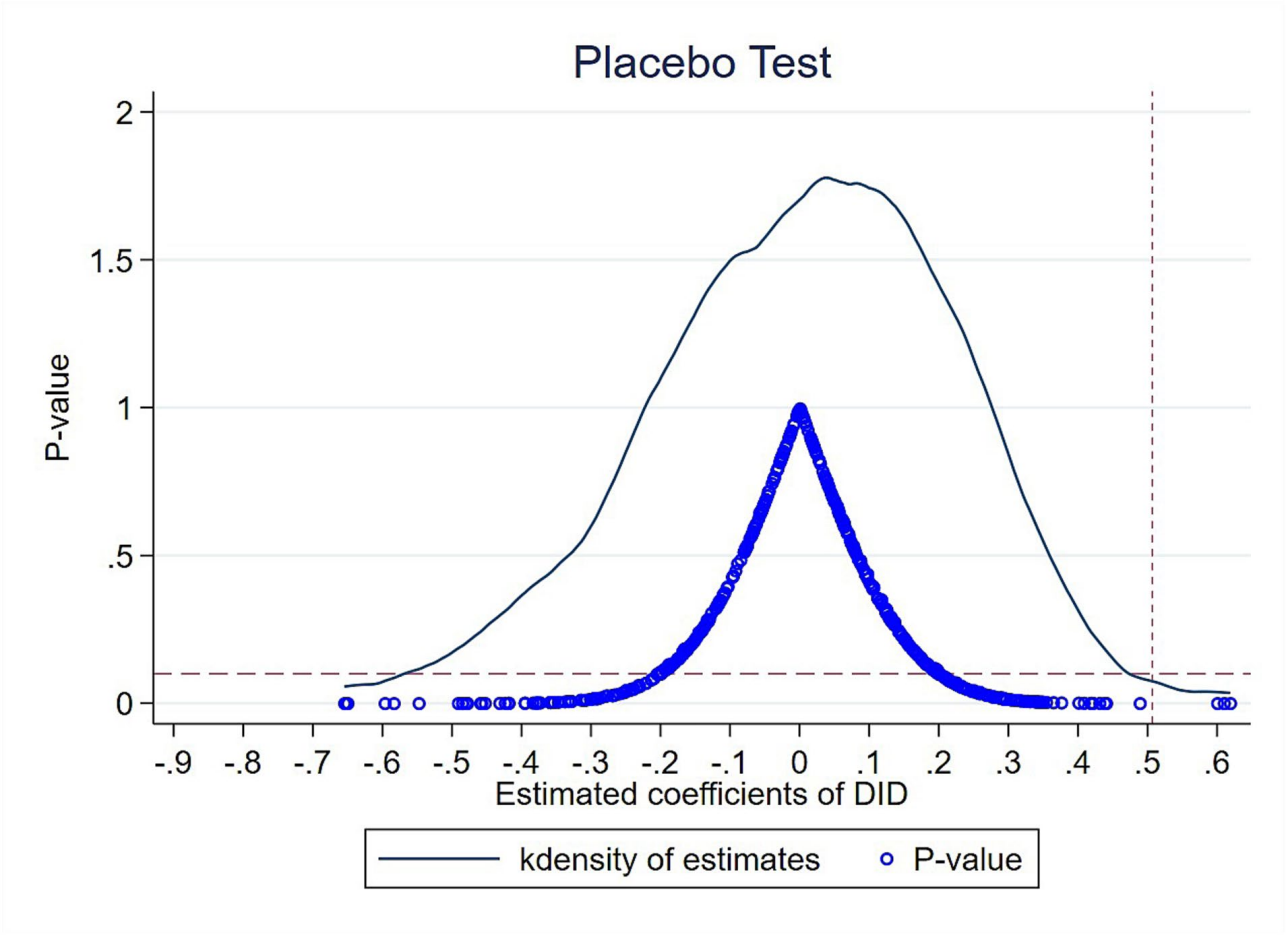
Placebo test.

The above empirical results demonstrate that there was no significant difference between the financial performance of the experimental and control groups before the NCDP policy, while the financial performance of the experimental group was significantly higher than that of the control group after the NCDP policy. To verify that this difference was caused by the NCDP policy and not by other policies or unobservable factors, a placebo test was used. In the experimental group, there were 20 bid-winning enterprises, so 20 enterprises were randomly selected from among all enterprises as the “pseudo- experimental group” (assuming that these enterprises were the bid-winning enterprises) and the remaining enterprises were used as the control group. “Pseudo-DID” variables were generated and the DID method was performed using the main DID regression model (2). Bootstrap sampling was used to resample 500 times to solve problems such as the lack of randomness of the sample. Figure 3 shows the distribution of the 500 estimated coefficients of the “pseudo-DID” variables, indicating that the coefficients generally conformed to a normal distribution with a mean of 0 and were far from the true DID model (2) estimate (0.507). In addition, most of the *p*-values were > 0.1, i.e., not significant at the 10% level. The results of the placebo test suggest that the study estimates were not obtained by chance and were unlikely to have been influenced by other policies or random factors.

### 4.3. Heterogeneity analysis

Although NCDP participation clearly improved financial performance, it was unknown whether NCDP participation had different impacts on bid-winning enterprises with different types of ownership. Therefore, the full sample was divided into two categories to examine whether there was a difference in the effect of the NCDP policy on the financial performance of SOEs and non-SOEs (Table 6). The results show that the DID regression coefficient for SOEs was not significant, while the DID regression coefficient for non-SOEs was significantly positive at the 1% level. This indicates that NCDP participation was more favorable for the financial performance of non-SOEs.

**Table 6:** Heterogeneity analysis of enterprise ownership.

Variable	SOEs	Non-SOEs
DID	−0.001 (0.239)	0.648*** (0.119)
Control variables	Yes	Yes
Enterprise fixed effects	Yes	Yes
Quarter fixed effects	Yes	Yes
adj. R^2^	0.326	0.715

## 5. Discussion

### 5.1. Interpretation of findings

The findings of this study suggest that NCDP participation by pharmaceutical enterprises improves their financial performance, corroborating the views of some researchers (16). For the empirical results, we offer the following interpretation from two dimensions: procurement mechanism and policy orientation.

The impact of NCDP on financial performance is mainly achieved through a procurement mechanism involving “volume–price linkage,” “volume for price,” and “price-based volume,” along with the policy direction of “promoting R&D innovation.”

First, the procurement mechanism enables bid-winning enterprises to expand their market share while reducing their production costs. From an economic viewpoint, this matches the concept of “selling more at lower prices,” with a large market share being gained through price concessions. Before the NCDP implementation, the production costs and reasonable profits only accounted for a relatively small portion of the drug prices. Large costs such as sales and marketing expenses caused excessive drug prices. For example, many pharmaceutical enterprises hired representatives to visit public medical institutions to promote their products in order to increase their market share. As there had previously been a long period of inflated drug prices in China, the price concessions made by the bid-winning enterprises allowed the drug prices to be guided by the market. The large volume of agreed purchases that enterprises get by making price concessions not only increases revenue, but also saves on costs related to advertising and promoting products at medical institutions. At the same time, thanks to strong policy advocacy, patient recognition and trust regarding the bid-winning drugs continue to grow, despite the bias among some patients against generics prior to NCDP implementation. In public medical institutions, the incentive mechanism related to the agreed usage of the bid-winning drugs and the practice of supporting medical insurance policies have both increased clinicians’ willingness to use bid-winning drugs (51). In the long run, the drug use structure of public medical institutions and the drug consumption habits of patients will further change, and the bid-winning enterprises will establish brand effects without spending money on advertising. In addition, it is worth noting that several of the bid-winning enterprises are leading companies with complete industrial chains, with simultaneous API, intermediate, and formulation production modules. This comprehensive production model is conducive to reducing procurement costs and improving financial performance.

Second, the implementation of the NCDP policy encourages enterprises to invest more in R&D innovation to improve their financial performance. It has been proven that continuous and stable R&D investment can enhance the core competitiveness and profitability of enterprises, thus contributing to the improvement of their financial performance (52, 53). Since the implementation of the NCDP policy, the trend of generic drugs replacing original drugs has become more and more evident. Generic drugs that have not passed the GCE will have little or no foothold in the market, so original drugs and high-quality generic drugs will dominate the market. As a large producer of generic drugs, China often produces drugs with excessive homogeneity. To ensure the continued competitiveness of their drugs and their continued participation in NCDP, enterprises will likely invest heavily in R&D. Generic drug manufacturers can invest in R&D by improving production efficiency and upgrading formulation processes, thereby reducing production costs, developing high-quality generic drugs, reducing inter-product substitutability, increasing competitive advantages, and improving financial performance. Moreover, due to the decreased profit margins of generic drugs, some enterprises will conduct R&D to be able to provide original drugs in order to increase their profitability.

Finally, the impact of NCDP on bid-winning enterprises varies depending on the ownership structure. NCDP participation considerably contributes to the financial performance of non-SOEs, but not SOEs. This result may be partly due to the fact that SOEs are more focused on sociopolitical goals than on maximizing corporate interests. For SOEs participating in the NCDP, which is a drug price reform that aims to benefit the whole society, bids are won not only for economic benefits, but also to fulfill a sociopolitical mission. As a result, when constrained by drug prices and agreed procurement volumes, SOEs face greater supply pressure, and tend to operate with the goal of ensuring supply volumes for social responsibility reasons, as well as to avoid administrative penalties. The resulting increased costs make it difficult for SOEs to improve their financial performance quickly. In contrast, compared to SOEs, non-SOEs are less constrained by social responsibility and are able to make production decisions independently and autonomously. The higher resource allocation efficiency of non-SOEs can also compensate for the economic losses caused by policy pressures, leading to faster financial performance improvement. Furthermore, we should recognize that the two enterprise types have different resources and are subject to different competitive pressures. SOEs have easier access to banks and other financial support, and the resources needed for their survival and development are relatively easy to obtain. Their sensitivity to competitive pressure is also relatively weak, so the strengthening effect of NCDP participation on financial performance is not significant. In contrast, non-SOEs face fierce competitive pressures in the market, and only by actively participating in NCDP and increasing their market share can they increase their chances of survival, so the strengthening effect of NCDP participation on financial performance is significant.

### 5.2. Policy insights

There are several policy insights from this research. First, this study demonstrates that NCDP participation has positive implications for the financial performance of bid-winning pharmaceutical enterprises. By September 2023, eight rounds of NCDP had been conducted in China, and a large number of clinically important chemical drugs are now included on the procurement list. To promote the transformation of the entire pharmaceutical industry and improve the quality of more drugs, the inclusion of traditional Chinese medicine and more biological drugs on the procurement list should be considered in the subsequent NCDP.

Second, the government should increase assistance to R&D-based pharmaceutical enterprises and provide more policy support. Currently, the protection of patented pharmaceutical technologies in China is not strong enough and the related legal system is imperfect, which allows competitors to more easily steal or exploit other enterprises’ technological innovations. In addition, most Chinese pharmaceutical enterprises’ R&D focuses on simple imitation and improvement, which is not conducive to long-term development. All these factors decrease the positive effect of the NCDP policy on financial performance. Therefore, policymakers should improve the innovation incentive mechanisms and the regulatory system to fully motivate enterprises to conduct R&D and thus improve their financial performance.

Finally, the government should improve the linkages between health care, health insurance, and the NCDP policy. Ensuring the use of bid-winning drugs in public medical institutions is an important part of pharmaceutical enterprises’ efforts to improve their financial performance. The National Medical Products Administration (NMPA) should also urgently promote GCE and conduct extensive clinician training and education to alter prescribing habits. Lastly, the National Health Insurance Administration should explore reforms to health insurance payment methods, such as including the bid-winning drugs in the diagnosis-related groups (DRG) disease treatment plans and increasing the reimbursement rates of the bid-winning drugs, so as to guarantee stable market shares for bid-winning enterprises.

### 5.3. Strengths and limitations

To the best of our knowledge, this is the first study to assess the impact of the NCDP policy on the financial performance of listed pharmaceutical enterprises using nationwide panel data. This study has the following strengths. First, the study covered listed pharmaceutical enterprises not only in 11 key cities (four municipalities and seven sub-provincial cities involved in the “4 + 7” procurement period), but the entire Chinese mainland, and the OGS, a comprehensive indicator, was used to assess financial performance. These approaches enabled this study to better reflect the impact of the NCDP policy on financial performance. Second, we viewed the implementation of the NCDP policy as a quasi-natural experiment and used the DID model in order to minimize the interference of unobservable factors in the results and improve the credibility of the results. We conducted parallel trend and placebo tests on the DID model and verified that the PSM-DID model results generally agreed with the main DID results, demonstrating the robustness of the results. Finally, we examined the differential effects of NCDP participation on the financial performance of SOEs and non-SOEs and discussed the potential causes.

Our study also has several limitations. First, the enterprises in our sample were all listed enterprises, but not all of the enterprises that participate in NCDP are listed enterprises, so the findings may not apply to all participating pharmaceutical enterprises. However, listed enterprises are important subjects not only for the pharmaceutical industry but also for the NCDP policy, and they have complete financial data, so the sample is useful for analyzing the impact of the NCDP policy on the pharmaceutical enterprises’ financial performance. Second, we constructed a counterfactual framework in the study. However, it is worth noting that most of the bid-winning enterprises are leading companies, and it was difficult to identify a control group of Chinese listed pharmaceutical enterprises that are highly comparable to the enterprises in the experimental group based on all financial metrics. Although the results of the parallel trend test supported the parallel trend assumption of the DID method, and the PSM method led to a suitable control group being constructed, there were still shortcomings in the establishment of the control group, so there may be some risk of bias in the results. Finally, there are still some factors that may have led to errors in our estimates of the effectiveness of NCDP participation. For example, during the study time period, the Chinese government also engaged in policy practices such as national drug negotiations, which may have had an impact on pharmaceutical enterprises’ financial performance. Therefore, the DID model and robust tests were used to minimize the impact of these policy practices on the results.

## 6. Conclusion

In this study, we evaluated the impact of the NCDP policy on corporate financial performance using the DID method, based on panel data from 174 listed pharmaceutical enterprises in China from the second quarter of 2018 to the fourth quarter of 2021, and we drew the following conclusions. Before the implementation of the NCDP policy, the difference in financial performance between enterprises in the experimental and control groups was not significant. After the implementation of the NCDP policy, the financial performance of the experimental group enterprises increased significantly. This indicates that NCDP participation has a positive impact on pharmaceutical enterprises’ financial performance. The impact stems from lower costs, increased market share, and increased R&D investment. In the future, the government may implement more policies such as including more drugs in NCDP, accelerating GCE, improving the incentive mechanisms for medical institutions to use the bid-winning drugs, and improving the drug patent protection system, so as to improve the accessibility and affordability of drugs while improving pharmaceutical enterprises’ financial performance.

## Data availability statement

Publicly available datasets were analyzed in this study. This data can be found at: https://csmar.zssgdsb-85176920tsgjnz.com/ China Stock Market & Accounting Research Database (CSMAR).

## Author contributions

SC and ZS: article conception and design. ZS: data collection and analysis and initial manuscript writing. XN: article editing and proofreading. All authors contributed to the article and approved the submitted version.
