# Supplementary material for: Impact of China’s National Centralized Drug Procurement Policy on pharmaceutical enterprises’ financial performance: a quasi-natural experimental study
Source: Front Public Health. 2023 Nov 3;11:1227102. doi: 10.3389/fpubh.2023.1227102 (PMC10654749; doi:10.3389/fpubh.2023.1227102)
Supplement: Supplementary file 1 [file Table_1.DOCX]

Supplementary Material

Impact of China’s National Centralized Drug Procurement Policy on Pharmaceutical Enterprises’ Financial Performance: A Quasi-Natural Experimental Study

**Zhixuan Sun, Xin Na, Shuzhen Chu^*^**

*** Correspondence: 1019941160@cpu.edu.cn**

# Supplementary Table

The table incorporates the specifics of the 174 listed pharmaceutical enterprises in the study sample, categorized into 20 bid-winners (experimental group) and 154 non-bid-winners (control group), including the listed company code, the enterprise's abbreviation, and the number of bid-winning drug varieties.

| Experimental group (bid-winning enterprises) | | | | |
| --- | --- | --- | --- | --- |
| Listed enterprise code | Enterprise abbreviation | | Number of bid-winning drug varieties | |
| 518 | Sihuan Bioengineering | | 1 | |
| 756 | Xinhua Pharmaceutical | | 2 | |
| 999 | China Resources Sanjiu | | 1 | |
| 2020 | Jingxin Pharmaceutical | | 1 | |
| 2294 | Salubris Pharmaceuticals | | 1 | |
| 2422 | Kelun Pharmaceutical | | 5 | |
| 2907 | Pharscin Pharmaceutical | | 2 | |
| 300016 | Beilu Pharmaceutical | | 1 | |
| 300199 | Hybio Pharmaceutical | | 1 | |
| 300436 | Cosunter Pharmaceutical | | 1 | |
| 300705 | Jiudian Pharmaceutical | | 1 | |
| 600079 | Humanwell Healthcare | | 2 | |
| 600227 | Salvage Pharmaceutical | | 1 | |
| 600267 | Hisun Pharmaceutical | | 1 | |
| 600276 | Hengrui Pharmaceuticals | | 3 | |
| 600521 | Huahai Pharmaceutical | | 2 | |
| 600535 | Tasly Pharmaceutical | | 2 | |
| 600572 | Conba Pharmaceutical | | 1 | |
| 600789 | Lukang Pharmaceutical | | 2 | |
| 600812 | North China Pharmaceutical | | 2 | |
| Control group (non-bid-winning enterprises) | | | | |
| Listed enterprise code | Enterprise abbreviation | Listed enterprise code | | Enterprise abbreviation |
| 153 | Fengyuan Pharmaceutical | 300110 | | Huaren Pharmaceutical |
| 403 | Pacific Shuanglin Bio-pharmacy | 300111 | | Sunflower Great Health |
| 423 | Dong-E-E-Jiao | 300122 | | Zhifei Biological |
| 513 | Livzon Pharmaceutical | 300142 | | Walvax Biotechnology |
| 534 | Wedge Industrial | 300147 | | Xiangxue Pharmaceutical |
| 538 | Yunnan Baiyao | 300158 | | Zhendong Pharmacy |
| 566 | Hainan Haiyao | 300181 | | Jolly Pharmaceutical |
| 590 | Tus-pharmaceutical | 300194 | | Fuan Pharmaceutical |
| 597 | Northeast Pharmaceutical | 300204 | | Staidson |
| 623 | Aodong Pharmaceutical | 300233 | | Jincheng Pharmaceutical |
| 650 | Renhe Pharmacy | 300239 | | Dongbao Bio-Tech |
| 661 | Changchun High-Tech | 300254 | | C&Y Group |
| 739 | Apeloa Pharmaceutical | 300255 | | Changshan Biochemical |
| 766 | Tonghua Golden-horse | 300267 | | Er-Kang Pharmaceutical |
| 788 | PKU HealthCare | 300294 | | Boya Bio-Pharmaceutical |
| 790 | Huasun Technology | 300357 | | Wolwo Bio-Pharmaceutical |
| 813 | Dezhan Healthcare | 300363 | | Porton Pharma |
| 908 | Jingfeng Pharmaceutica | 300434 | | Goldstone Asia Pharmaceutical |
| 915 | Wit Dyne | 300485 | | Science Sun Pharmaceutical |
| 919 | Jinling Pharmaceutical | 300497 | | Fushine Pharmaceutical |
| 931 | Centergate Technologies | 300519 | | Xinguang Pharmaceutical |
| 952 | Guangji Pharmaceutical | 300534 | | Longshenrongfa |
| 989 | Jiuzhitang | 300558 | | Betta Pharmaceuticals |
| 2001 | Zhejiang NHU | 300573 | | Xingqi Pharmaceutical |
| 2007 | Hualan Biological | 300584 | | Hicin Pharmaceutical |
| 2019 | Yifan Pharmaceutical | 300630 | | Poly Pharm |
| 2038 | SL Pharmaceutical | 300636 | | Synergy Pharmaceutical |
| 2099 | Hisoar Pharmaceutical | 300683 | | Hiteck biopharmaceutical |
| 2107 | Wohua Pharmaceutical | 300702 | | Tianyu Pharmaceutical |
| 2118 | Zixin Pharmaceutical | 300723 | | ApicHope |
| 2166 | Layn Natural Ingredients | 600062 | | China Resources Double-crane |
| 2198 | Jiaying Pharmaceutical | 600085 | | Tongrentang |
| 2252 | RAAS Blood Products | 600129 | | Taiji Industry |
| 2262 | Nhwa Pharmaceutical | 600161 | | tiantan biological |
| 2275 | Sanjin Pharmaceutical | 600196 | | Fosun Pharma |
| 2287 | Cheezheng Tibetan Medicine | 600211 | | Rhodiola Pharmaceutical |
| 2317 | Zhongsheng Pharmaceutical | 600216 | | Zhejiang Medicine |
| 2332 | Xianju Pharmaceutical | 600222 | | Taloph Pharmaceutical |
| 2349 | Jinghua Pharmaceutical | 600252 | | Zhongheng Group |
| 2365 | Yongan Pharmaceutical | 600285 | | Lingrui Pharmaceutical |
| 2370 | Yatai Pharmaceutical | 600329 | | Zhongxin Pharmaceutical |
| 2390 | Xinbang Pharmaceutical | 600332 | | Baiyunshan |
| 2393 | Lisheng Pharmaceutical | 600351 | | Yabao Pharmaceutical |
| 2399 | Hepalink Pharmaceutical | 600380 | | Joincare Pharmaceutical |
| 2412 | Hansen Pharmaceutical | 600420 | | Shyndec Pharmaceutical |
| 2424 | Guizhou Bailing | 600422 | | KPC Pharmaceuticals |
| 2435 | Changjiang Health | 600436 | | Pien Tze Huang |
| 2437 | Gloria Pharmaceuticals | 600479 | | QianJin Pharmaceutical |
| 2550 | Qianhong Biopharma | 600488 | | Tianyao Pharmaceuticals |
| 2562 | Brother Enterprises | 600513 | | Lianhuan Pharmaceutical |
| 2566 | Yisheng Pharmaceutical | 600557 | | Kanion Pharmaceutical |
| 2581 | Sinobioway Biomedicine | 600566 | | Jumpcan Pharmaceutical |
| 2603 | Yiling Pharmaceutical | 600594 | | Yibai Pharmaceutical |
| 2644 | Foci Pharmaceutical | 600613 | | Shenqi Pharmaceutical |
| 2653 | Haisco Pharmaceutical | 600664 | | Harbin Pharmaceutical |
| 2675 | Dongcheng Pharma | 600750 | | Jiangzhong Pharmaceutical |
| 2693 | Shuangcheng Pharmaceuticals | 600771 | | GuangYuYuan |
| 2728 | Teyi Pharmaceutical | 600851 | | Haixin Group |
| 2737 | Sunflower Pharmaceutical | 600867 | | Tonghua Dongbao |
| 2750 | Longjin Pharmaceutical | 603139 | | Kanghui Pharmaceutical |
| 2755 | Aosaikang Pharmaceutical | 603168 | | Shapuaisi Pharma |
| 2773 | Kanghong Pharmaceutical | 603222 | | Chimin Health |
| 2793 | Luoxin Pharmaceuticals | 603229 | | Ausun Pharmaceutical |
| 2817 | Huangshan Capsule | 603367 | | Cisen Pharmaceutical |
| 2821 | Asymchem Laboratories | 603456 | | Jiuzhou Pharmaceutical |
| 2826 | Tibet Aim Pharm | 603520 | | Starry Pharmaceutical |
| 2864 | Panlong Pharmaceutical | 603538 | | Menovo Pharmaceutical |
| 2873 | Xintian Pharmaceutical | 603567 | | ZBD Pharmaceutical |
| 2880 | Weiguang Biological | 603669 | | Lionco Pharmaceutical |
| 2898 | Sailong Pharmaceutical | 603676 | | Weixinkang Pharmaceutical |
| 2900 | Harbin Medisan | 603707 | | King-Friend Biochemical |
| 2923 | Rundu Pharmaceutical | 603811 | | Cheng Yi Pharmaceutical |
| 2932 | Easy Diagnosis | 603858 | | Buchang Pharmaceuticals |
| 300006 | Lummy Pharmaceutical | 603880 | | Nanfang Medical |
| 300009 | Anke Biotechnology | 603896 | | Shouxiangu Pharmaceutical |
| 300026 | Chase sun Pharmaceutical | 603963 | | Dali Pharmaceutical |
| 300039 | Kaibao Pharmaceutical | 603998 | | Fangsheng Pharmaceutical |
| 300086 | Honz Pharmaceutical |  | |  |
